# Same‐day antiretroviral therapy initiation hub model at the Thai Red Cross Anonymous Clinic in Bangkok, Thailand: an observational cohort study

**DOI:** 10.1002/jia2.25869

**Published:** 2021-12-30

**Authors:** Pich Seekaew, Nittaya Phanuphak, Nipat Teeratakulpisarn, Sorawit Amatavete, Sita Lujintanon, Somsong Teeratakulpisarn, Tippawan Pankam, Oranuch Nampaisan, Pintip Jomja, Chotika Prabjunteuk, Prapaipan Plodgratoke, Reshmie Ramautarsing, Praphan Phanuphak

**Affiliations:** ^1^ Department of Epidemiology Columbia University Mailman School of Public Health New York New York USA; ^2^ Institute of HIV Research and Innovation Bangkok Thailand; ^3^ Thai Red Cross AIDS Research Centre Bangkok Thailand

**Keywords:** treatment, key and vulnerable populations, linkage to care, retention, viral suppression

## Abstract

**Introduction:**

WHO has recommended rapid antiretroviral therapy (ART) initiation, including same‐day ART (SDART). However, data on the feasibility in real‐world settings are limited. We implemented a cohort study at a stand‐alone HIV testing centre to examine its applicability and effectiveness.

**Methods:**

Data were collected from the Thai Red Cross Anonymous Clinic in Bangkok, Thailand, between July 2017 and July 2018 from clients who were ART‐naïve and could return for follow‐up visits. Baseline laboratory tests and chest X‐ray were performed according to national guidelines, and clinical eligibility was determined based on physical examination and chest X‐ray findings. Primary outcomes were retention in care and viral load suppression at 3, 6 and 12 months.

**Results:**

During the study period, 2427 people tested HIV positive. Of these, 2107 (2207/2427, 86.8%) met logistical criteria, and 1904 (1904/2427, 78.5%) agreed to SDART. One thousand seven hundred and twenty‐nine (1729/2427, 71.2%) were placed on ART, with 1257 received same‐day initiation and 1576 initiated ART within 7 days; 1198 clients were successfully referred to free, sustained ART sites. Retention among eligible clients who accepted SDART service at months 3, 6 and 12 was 79.8%, 75.2% and 75.3%, respectively.

**Conclusions:**

Same‐day ART initiation hub model at a stand‐alone HIV testing centre in an urban setting in Bangkok, Thailand, is highly feasible and has a potential for scaling up.

**Clinical Trial Number:**

NCT04032028

## 1 INTRODUCTION

Recent data show that early initiation of antiretroviral therapy (ART) benefits people living with HIV (PLHIV) and reduces the risk of HIV transmission to others [1, 2, 3]. These data form the basis for 2015 the World Health Organization (WHO) treatment guidelines that ART should be initiated at any CD4 level [4]. Despite this recommendation, many PLHIV are not on treatment, particularly in resource‐constrained settings [5, 6, 7]. Barriers at multiple levels contribute to this phenomenon, including pre‐treatment attrition [7, 8, 9]. Additionally, multiple preparatory visits and lengthy assessment and treatment preparedness processes can further delay treatment initiation, which has negative effects on treatment initiation rates and health outcomes [10, 11, 12, 13]. Men who have sex with men (MSM) and transgender women (TGW) are even less likely than the general population to start treatment, which may be due to barriers they face in accessing medical services as sexual minorities [14, 15, 16]. In 2018, it was estimated that 12–15% of Thai MSM and 11% of Thai TGW were living with HIV, and these populations contributed to almost half of all new HIV infections in the country [17]. Thus, it is imperative to evaluate and optimize HIV and sexual health services that accommodate these populations.

Accessibility to ART has been a challenge in Thailand. In 2016, Bangkok represented one‐third of total new infections nationally, with approximately 47,000 PLHIV, but only 57% received ART [18]. Moreover, only 79% of those who had received treatment were virally suppressed [18], leaving the rest at increased health risk and vulnerable to transmitting the virus. Most HIV testing in Bangkok occurs at testing facilities rather than hospitals, where ART initiation traditionally happens. Linking PLHIV from a testing site to their preferred long‐term ART hospital has proven difficult because doing so requires multiple steps, depending on the individual PLHIV's health benefit scheme. These delays can jeopardize the health of those without treatment and pose a serious public health threat due to the potential for onward transmission.

New evidence demonstrates the potential benefits of offering rapid ART initiation, including increased successful links to care and ART uptake [19, 20, 21, 22, 23]. The WHO has also recommended same‐day ART (SDART) initiation [24]. However, there have been no published investigations of SDART initiation models operating from Asian settings. Given that HIV testing is increasingly occurring in community settings, the addition of SDART to these settings offers an opportunity to reduce loss to follow‐up and accelerate ART initiation. This manuscript describes the implementation and treatment outcomes of a “Same‐Day ART Initiation Hub” model at the Thai Red Cross Anonymous Clinic (TRC‐AC) in Bangkok, Thailand. The primary outcomes are retention in care and viral load suppression 3, 6 and 12 months after ART initiation.

## 2 METHODS

### 2.1 Study design and participants

This is an open‐ended, prospective, observational cohort study enrolling clients who have tested HIV positive at TRC‐AC in Bangkok, Thailand. As a stand‐alone HIV‐testing centre, TRC‐AC offers sexual health services, including HIV and sexually transmitted infections testing, pre‐exposure prophylaxis, post‐exposure prophylaxis, and vaginal, anal and neovaginal pre‐cancerous screening services. ART and other medications are available for purchase, and health benefit schemes cannot reimburse them; clients must go to their assigned hospital based on their health benefit scheme to receive free ART. This study included clients who tested HIV positive between July 2017 and July 2018 and were followed up until July 2019.

SDART was introduced at TRC‐AC in July 2017, which allows clients who are confirmed to have HIV to initiate ART, if they are willing and deemed clinically eligible, before being referred out to their respective assigned hospital. Individuals who are newly diagnosed HIV positive at TRC‐AC and individuals with prior HIV diagnosis but first re‐engaged in care at TRC‐AC (re‐engaged client) are eligible to participate. Additionally, peer‐navigators, most of whom are key populations, are recruited to provide continuous psychosocial and emotional support to our clients and assist the clients with hospital referrals.

### 2.2 Procedures

Eligible clients were assessed by a counsellor for willingness to access SDART service following receipt of a reactive anti‐HIV screening test using Architect HIV Ag/Ab Combo (Abbott, Wiesbaden, Germany) or Elecsys HIV combi PT (Roche Diagnostic GmbH, Mannheim, Germany). Those who consented received phlebotomy to conduct two additional anti‐HIV tests for confirmation using WANTAI RAPID TEST (Beijing Wantai Biological Pharmacy Enterprise Co., Ltd., Beijing, China) and SERODIA® HIV‐1/2 (Fujirebio Inc., Tokyo, Japan) (following the national diagnostic algorithm), as well as additional baseline pre‐ART laboratory tests. Baseline laboratory and clinical assessment, following Thailand's national ART guidelines [25], included complete blood count, creatinine, alanine aminotransferase, urinalysis, CD4 count, hepatitis B surface antigen (HBsAg), hepatitis C antibody (anti‐HCV), syphilis serology, chest X‐ray and cryptococcal antigen (only if CD4 count <100 cells/mm^3^). All clients received chest X‐ray irrespective of TB‐indicative symptoms. Clients were also evaluated based on TB‐indicative symptoms and chest X‐ray findings to determine if GeneXpert MTB/RIF testing was necessary. After receiving the results from confirmatory anti‐HIV tests and chest X‐ray, a nurse and a non‐specialist physician collected a medical history. They performed a physical examination to rule out potential opportunistic infections, including tuberculosis and cryptococcal meningitis.

All clients who were enrolled in SDART received initial and follow‐up support from a “peer navigator,” whose responsibilities included adherence support during the ART initiation period and linkage to long‐term care after ART initiation. Navigators were themselves MSM, TGW, heterosexual women and/or PLHIV. During the first month after SDART initiation, navigators helped clients schedule an appointment with a primary care provider at their preferred long‐term ART site and accompanied clients to their first visit. SDART service was free‐of‐charge to all clients, including ART, baseline tests and chest X‐ray.

Clients with no suspected illnesses were initiated ART during this first visit and were supplied with a free, 2‐week ART supply and asked to come back for a follow‐up visit in 14 days. The standard first‐line ART regimen was tenofovir disoproxil fumarate (TDF) 300 mg, emtricitabine (FTC) 200 mg and efavirenz (EFV) 600 mg once daily. This is the preferred regimen per National Guidelines during the study period. Clients with baseline creatinine clearance <60 ml/minute, as calculated by Cockcroft–Gault equation, were contacted to come back as soon as possible prior to their scheduled visit to have TDF/FTC switched to zidovudine (AZT) 300 mg plus lamivudine (3TC) 150 mg twice a day. Those with any other abnormal laboratory results were assessed for further investigations and ART regimen modifications as needed. Those with normal laboratory results were notified of all results during the 2‐week follow‐up visit and were assessed for potential ART‐related side effects and ART adherence. If clients had no contraindication, ART was provided for another 2 months to allow for adequate ART supply during their long‐term ART maintenance site transition. ART initiation was delayed for clients with medical contraindications, and these clients were referred to a hospital for further investigation with follow‐up support provided by the navigators.

Finally, clients who declined SDART, were not ART‐naïve or could not return in 2 weeks received same‐day confirmatory testing according to the national algorithm (but no additional baseline testing) and were referred to a hospital‐based ART provider of their preference following standard protocols. Retention and treatment outcomes were assessed 3, 6 and 12 months after ART initiation.

### 2.3 Outcome measures

Primary endpoints were retention in care and viral load (VL) suppression. Retention in care was defined as being in contact with and refilling antiretroviral therapy (ART) at a preferred ART site 3, 6 and 12 months after ART initiation. Retention was assessed by calling clients and checking their ART status on a national HIV database system website, NAPPLUS (http://dmis.nhso.go.th/NAPPLUS/login.jsp), for those who were under National Health Security Office and Social Security Office health benefit schemes. Clients who could not be contacted via phone calls and no data were registered on the NAPPLUS website 1 month after each specified timepoint was considered lost to follow‐up (LTFU). VL suppression was defined as having HIV‐1 RNA <50 copies/ml 6 and 12 months after ART as assessed through the NAPPLUS website. Only viral load results documented on NAPPLUS website were included in the analysis. Secondary endpoints were acceptability of the program, time to ART initiation and survival. Acceptability was evaluated according to the number of clients who agreed to enter the SDART service when offered. The duration between HIV diagnosis to care engagement was defined as the first time knowing HIV‐positive status (for a re‐engaged client) or having the first reactive anti‐HIV test result (for a newly diagnosed client) to seeing a navigator (entry point of SDART service). Time to ART initiation was measured as the time from seeing navigators (care engagement) to ART initiation; deaths were ascertained by a report from a family member or listed as deceased on the NAPPLUS website. Data from clients who were excluded from SDART due to clinical contraindications were also collected to evaluate illness confirmation and treatment.

### 2.4 Statistical analysis

Characteristics at enrollment of clients who met SDART service inclusion criteria were summarized using frequency, simple proportions, mean (standard deviations, SDs) and medians (interquartile ranges, IQRs), according to the nature of the variables stratified by accepting the service. To compare the differences in outcomes between groups, Chi‐square or Fisher's exact test was used for categorical variables as appropriate, and Kruskal–Wallis test was used for continuous variables.

To evaluate the effectiveness of SDART service, data at months 3, 6 and 12 on retention in care and VL suppression were tabulated and stratified by populations. Data retrieved from July 2017 to July 2019 were included in the analyses. Statistical analysis was performed with Stata version 14 (Statacorp, College Station, TX).

### 2.5 Ethical consideration

This research project was approved by the Human Research Ethics committee at Chulalongkorn University (IRB:158/56.) The study is registered at Clinicaltrials.gov under identifier NCT04032028. Each client was provided the information statement and informed consent explaining the nature of the study, including methods and possible benefits and risks. Those who decided to participate gave verbal consent. Clients could refuse or withdraw to participate at any time and would be referred to an appropriate support group for treatment continuation.

## 3 RESULTS

**Figure 1:**
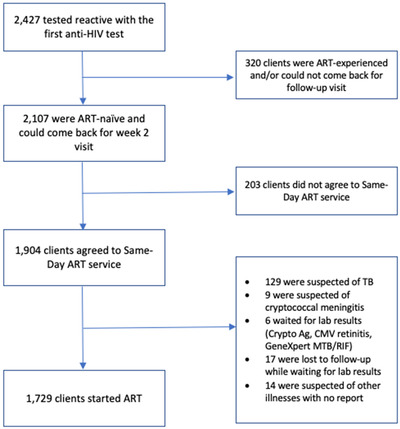
Flow of same‐day ART service at the Thai Red Cross Anonymous Clinic. All clients screened reactive for anti‐HIV were confirmed with two additional confirmatory anti‐HIV tests. Abbreviations: ART, antiretroviral therapy; CMV, cytomegalovirus; Crypto Ag, cryptococcal antigen; TB, tuberculosis.

**Figure 2:**
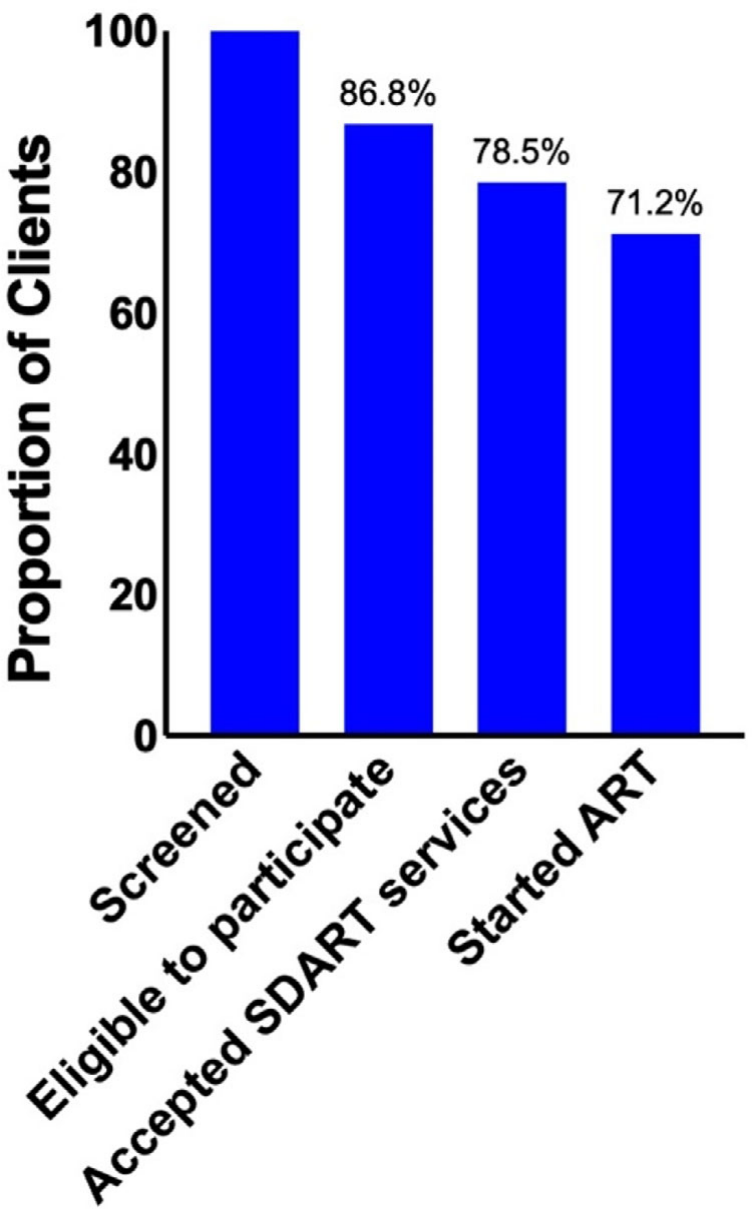
Same‐day ART service cascade and time to ART initiation. Same‐day ART service cascade with total clients (*n* = 2427) as the denominator.

From 1 July 2017 to 31 July 2018, 2427 people tested reactive with the first anti‐HIV screening, and 2107 (86.8%) were ART‐naïve and could come back for a 2‐week visit (Figure 1). Of these, 1904 (90.4% or 78.5% of 2427) agreed to SDART service before being clinically evaluated (Figure 2). Among those who agreed, 1112 (58.4%) were newly diagnosed. The acceptability among MSM, TGW and heterosexual client populations was 81.5%, 83% and 70.3%, respectively. The median (IQR) age of the clients was 28.3 years old (23.8–35.1) (Table 1).

**Table 1:** Demographic and clinical characteristics of clients who accepted same‐day ART service

	Total	Heterosexual	MSM	TGW	*p*‐Value
**Types of clients**					<0.001
*n*	1904	477	1334	93	
Newly diagnosed	1112 (58.4)	228 (47.8%)	839 (62.9%)	45 (48.4%)	
Re‐engaged	792 (41.6)	249 (52.2%)	495 (37.1%)	48 (51.6%)	
**Age at enrolment (years)** ^a^					<0.001
*n*	1853	470	1290	93	
Median (IQR)	28.3 (23.8‐35.1)	34 (27.5–41)	27.1 (23.2–32.9)	26.2 (23.3–29.5)	
**Age group** ^a^					<0.001
*n*	1853	470	1290	93	
<25 years old	584 (31.5%)	71 (15.1%)	477 (37%)	36 (38.7%)	
≥25 years old	126 (68.5%)	399 (84.9%)	813 (63%)	57 (61.3%)	
**Health benefit schemes** ^a^					<0.01
*n*	1900	476	1331	93	
NHSO	1013 (53.3%)	268 (56.3%)	682 (51.2%)	63 (67.7%)	
SSO	795 (41.8%)	182 (38.2%)	583 (43.8%)	30 (32.3%)	
CSS	92 (4.8%)	26 (5.5%)	66(5.0%)	0 (0.0%)	
**Duration from HIV diagnosis to care engagement (days)** ^a^					<0.001
*n*	1876	467	1317	92	
Median (IQR)	2 (0–8)	2 (0–11)	1 (0–7)	3 (1–7.5)	
**Time to ART initiation** ^a^					0.84
*n*	1729	413	1230	86	
Same day	1257 (72.7%)	287 (69.5%)	903 (73.4%)	67 (77.9%)	
1–2 days	156 (9%)	40 (9.7%)	109 (8.9%)	7 (8.1)	
3–7 days	163 (9.4%)	44 (10.7%)	112 (9.1%)	7 (8.1)	
8–14 days	32 (1.9%)	9 (2.2%)	22 (1.8%)	1(1.2)	
15–30 days	46 (2.7%)	10 (2.4%)	35 (2.8%)	1 (1.2)	
>30 days	75 (4.3%)	23 (5.6%)	49 (4.0%)	3 (3.5)	
**CD4 count (cells/mm^3^) (for those who started ART only)** ^a^					0.351
*n*	1694	401	1207	86	
Median (IQR)	294 (192–414)	288 (181–424)	294 (197–412)	313 (246–440)	
**CD4 count groups (for those who started ART only)** ^a^					0.06
*n*	1694	401	1207	86	
<200 cells/mm^3^	445 (26.3%)	122 (30.4%)	306 (25.4%)	17 (19.8%)	
200–499 cells/mm^3^	1015 (60.0%)	217 (54.1%)	743 (61.6%)	55 (64.0%)	
≥500 cells/mm^3^	234 (13.8%)	62 (15.5%)	158 (13.1%)	14 (16.3%)	
**CDC stage (for those who started ART only)** ^a^					0.81
*n*	1225	284	883	58	
A	1073 (87.6%)	245 (86.3%)	777 (88%)	51 (87.9%)	
B	135 (11.0%)	35 (12.3%)	93 (10.5%)	7 (12.1%)	
C	17 (1.4%)	4(1.4%)	13 (1.5%)	0 (0.0%)	

Abbreviations: ART, antiretroviral therapy; CDC, Centers for Disease Control and Prevention; CSS, Civil Servants Scheme; IQR, interquartile range; MSM, men who have sex with men; NHSO, National Health Security Office; SDART, same‐day antiretroviral therapy service; SSO, Social Security Office; TGW, transgender women.

^a^There were 51 cases with unspecified age, 4 cases with unspecified health benefit schemes, 28 cases with unspecified duration from HIV diagnosis to care engagement, 175 cases with unspecified time to ART initiation, 210 cases with unspecified CDC count and 679 cases with unspecified CDC stage.

**Figure 3:**
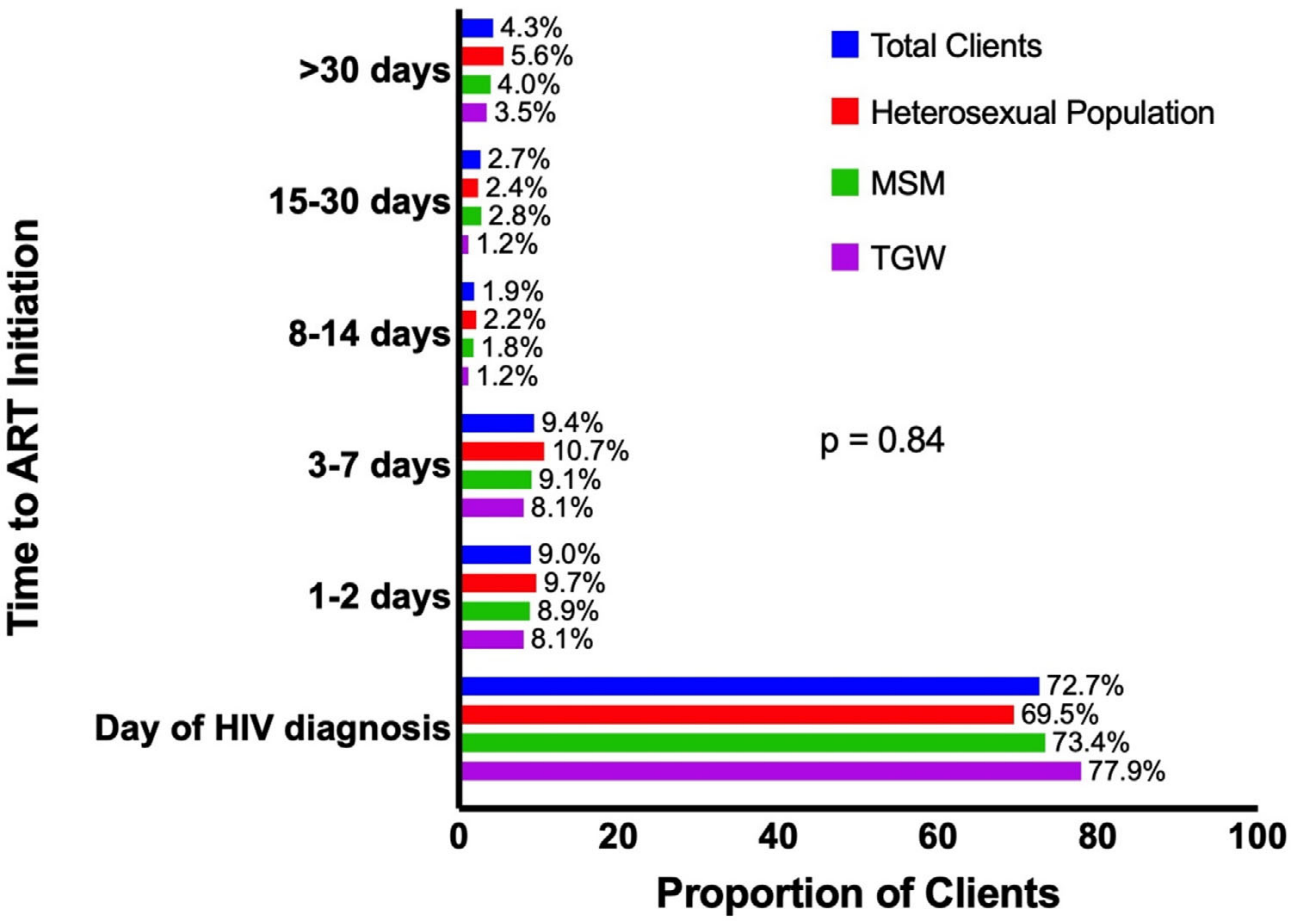
Time to ART initiation among eligible clients who accepted ART. Note: Time to ART initiation was measured as the time from seeing navigators and receiving confirmatory anti‐HIV results (care engagement) to ART initiation. The denominator was all eligible clients who accepted ART (*n*= 1729). The *p*‐value assessed the difference in duration of time to ART initiation between MSM, TGW and heterosexual clients. Abbreviations: MSM, men who have sex with men; TGW, transgender women.

The overall median (IQR) duration from HIV diagnosis to care engagement was 2 days (0–8). Of 1904 clients who accepted SDART service, 1729 (90% or 71.2% of 2427) met the clinical eligibility and were placed on ART. Of those who started ART in the SDART cohort, 72.7% (1257/1729) received same‐day initiation, with a total of 91.2% (1576/1729) started ART within 7 days (Figure 3). The overall median (IQR) CD4 count among clients who initiated ART was 294 (192–415) cells/mm^3^, with TGW having the highest median (IQR) CD4 count of 323.5 (262–442) cells/mm^3^ (Table 1).

Of 1292 clients who needed assistance with a referral, 92.8% (1199/1292) were successfully referred to their free preferred long‐term ART sites, with the median (IQR) time of 57 (39–70) days.

**Figure 4:**
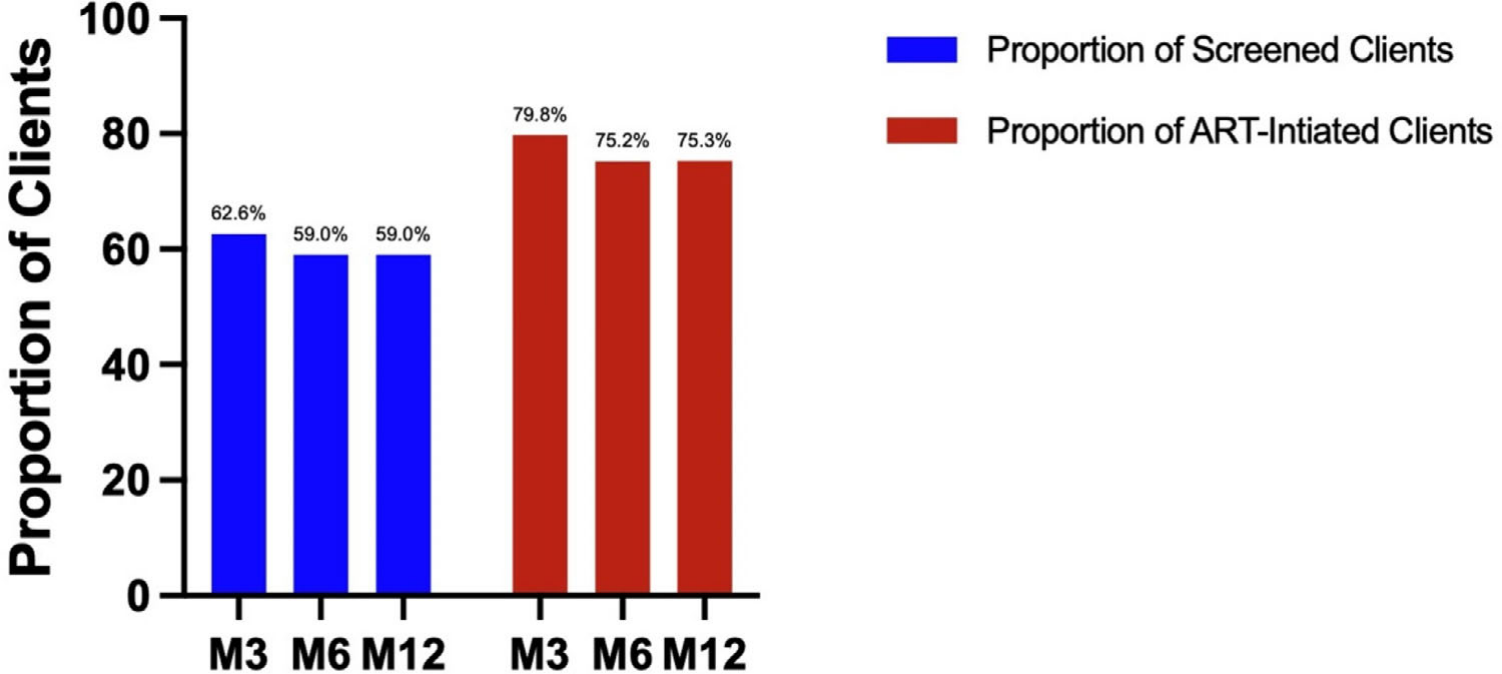
Retention. Note: Screened clients were individuals who tested positive with the first anti‐HIV test (*n*=2427). Information regarding the screened clients is provided in Table A1. ART‐initiated clients were eligible individuals who accepted SDART service and initiated ART (*n*=1904 for month 3; *n*=1899 for month 6; and *n*=1898 for month 12). Abbreviations: M3, month 3; M6, month 6; M12, month 12.

When evaluating from the total clients who tested positive, retention at months 3, 6 and 12 was: 62.6% (1520/2427), 59.0% (1428/2422) and 59% (1429/2421), respectively (Figure 4). Detailed information regarding long‐term outcomes among all clients who tested HIV positive at baseline is provided as an Appendix (Table A1). Among the clients who accepted SDART service, retention at months 3, 6 and 12 was: 79.8% (1520/1904), 75.2% (1428/1899) and 75.3% (1429/1898), respectively (Figure 4 and Table 2).

**Table 2:** Retention and viral suppression at 3, 6 and 12 months after ART initiation among clients who accepted same‐day ART service

	Total	Heterosexual	MSM	TGW	*p*‐Value
**Clients on ART for 3 months** ^a^					
*n*	1904	477	1334	93	
Retained in care	1520 (79.8%)	361 (75.7%)	1085 (81.3%)	74 (79.6%)	0.785
Lost to follow‐up	366 (19.2%)	109 (22.9%)	239 (17.9%)	18 (19.4%)	0.064
Discontinued ART	13 (0.7%)	5 (1.0%)	7 (0.5%)	1 (1.1%)	0.254
Death	5 (0.3%)	2 (0.4%)	3 (0.2%)	0 (0.0%)	0.694
**Clients on ART for 6 months** ^a^					
*n*	1899	475	1331	93	
Retained in care	1428 (75.2%)	336 (70.7%)	1021 (76.7%)	71 (76.3%)	0.792
Lost to follow‐up	456 (24%)	133 (28%)	301 (22.6%)	22 (23.7%)	0.073
Discontinued ART	14 (0.7%)	6 (1.3%)	8 (0.6%)	0 (0.0%)	0.252
Death	1 (0.1%)	0 (0.0%)	1 (0.1%)	0 (0.0%)	>0.99
**Clients on ART for 12 months** ^a^					
*n*	1898	475	1330	93	
Retained in care	1429 (75.3%)	330 (69.5%)	1028 (77.3%)	71 (76.3%)	0.329
Loss to follow‐up	460 (24.2%)	141 (29.7%)	297 (22.3%)	22 (23.7%)	0.006
Discontinued ART	8 (0.4%)	4 (0.8%)	4 (0.3%)	0 (0.0%)	0.238
Death	1 (0.1%)	0 (0.0%)	1 (0.1%)	0 (0.0%)	>0.99
**Viral suppression** ^a^					
*n*	1904	477	1334	93	
Overall viral suppressed	1214 (63.8)	292 (61.2)	863 (64.7)	59 (63.4)	0.69
Numbers of VL testing (within the first year)	1297 (68.1%)	309 (64.8%)	923 (69.2%)	65 (69.9%)	
Viral suppressed (within the first year)	1197 (62.9%)	288 (60.4%)	850 (63.7%)	59 (63.4%)	>0.99
Viral suppressed (within the first year and among VL‐tested clients)	1197/1297 (92.3%)	288/309 (93.2%)	850/923 (92.1%)	59/65 (90.8%)	

Abbreviations: ART, antiretroviral therapy; IQR, interquartile range; MSM, men who have sex with men; TGW, transgender women; VL, viral load.

^a^Retention and viral load suppression were assessed among clients who accepted SDART service, including clients who were referred out to a hospital for further clinical investigation.

Among clients who received ART in SDART service, 68.1% (1297/1904) were tested for VL within the first year. Of these, 92.3% (1197/1297) were virally suppressed. However, when assessing from the overall number of ART‐initiated clients, 62.9% (1197/1904) were virally suppressed (Table 2). The median (IQR) days from ART initiation to VL suppression was 200 (163–267).

## 4 DISCUSSION

We demonstrated that implementing SDART initiation hub model in Bangkok, Thailand, was acceptable among the MSM, TGW and heterosexual populations. Of the clients who tested HIV positive at baseline, 62.6%, 59% and 59% were retained in care at months 3, 6 and 12, respectively. However, among those who were eligible for and accepted SDART service, retention rates were 79.8%, 75.2% and 75.3% at 3, 6 and 12 months, respectively. Only 68.1% of the clients who initiated ART received VL testing; 92.3% were virally suppressed within the first year. Of the total clients who tested HIV positive at baseline, 53.4% received VL testing; 49.3% were virally suppressed. Among those who initiated ART in SDART service, we referred 92.8% of these clients to their preferred free long‐term ART maintenance site.

Our study also suggests that SDART may incentivize previously diagnosed individuals to seek treatment, as seen by the large proportion of re‐engaged clients in our cohort (41.6%). Furthermore, the results indicate that SDART can be safely implemented using a nonspecialized and simplified algorithm. Our flow uses symptomatic screening and chest X‐ray results to identify medical contraindications that would prompt treatment delay, while clients are notified of standard baseline laboratory results after treatment has already been initiated. Moreover, physicians prescribing ART were not specialists, suggesting that any physician with the knowledge of HIV treatment can initiate ART. Similar findings were also seen in the RapIT study, in which the authors suggested that laboratory results may not be needed in asymptomatic clients, reducing the need for point‐of‐care technology [21]. This increases the scalability of SDART to wider settings, expanding the potential beneficiaries who could be served under this model.

Despite high acceptability, 9.2% of the clients who agreed to the SDART service were excluded due to clinical concerns, with the majority being suspected of having TB (73.7%). This is of our interest, as TB is one of the significant contraindications that, if confirmed, ART initiation should be delayed. However, symptomatic screening through conventional TB‐indicative signs and symptoms has low sensitivity and low specificity [26], leading to an over‐exclusion of otherwise clinically eligible clients. This issue was also noted in a study conducted in South Africa and Kenya [27]. Moreover, though GeneXpert MTB/RIF has been recommended, it may be difficult to draw a conclusive result if the sputum is not obtained adequately, especially in a non‐hospital setting. Another tool that may be beneficial is lateral‐flow urine lipoarabinomannan assay (LF‐LAM), a non‐molecular, point‐of‐care test. Findings from the SLATE II trial found using LF‐LAM among individuals who reported TB‐indicative symptoms could help increase the number of ART eligible clients, rather than using symptomatic screening alone. By using this algorithm, the SLATE II study was able to initiate ART on more than 90% of nonpregnant adult clients within 7 days and showed that almost 90% of the clients were eligible for SDART initiation [28]. Although LF‐LAM has been demonstrated to be helpful in SLATE II, LF‐LAM is sensitive when a person has signs and symptoms of TB, with CD4 count less than or equal to 100 cells/mm^3^ [29]. Thus, it is not certain if LF‐LAM would be as applicable in our setting since the median CD4 among our clients was 294 cells/mm^3^. As more immediate ART initiation programs are being implemented, it is essential to have a simplified, rapid and sensitive screening tool to ensure feasibility in diverse settings. It is also important to note that the WHO recently issued updated guidelines recommending immediate ART initiation in non‐meningeal TB patients, regardless of CD4 count, and no longer than 2 weeks after TB treatment initiation [30].

Lengthy treatment readiness procedures are reported to be a significant factor delaying ART initiation, which also attributes to pre‐ART LTFU [7, 8, 9, 10, 11, 12, 13]. Our model illustrates that reducing wait times to start treatment had no adverse consequence on retention rates or viral suppression, which aligns with what has been found previously [20, 21]. Randomized trials conducted in Haiti and South Africa showed improved retention rates with VL suppression among those who had SDART initiation compared to the standard care arm [20, 21]. Additionally, a home‐based randomized clinical trial conducted in Lesotho showed a higher proportion of retention 12 months after ART initiation in the SDART arm when compared to the standard of care arm (63.5% vs. 48.2%, respectively; *p* = 0.01) [19]. However, an observational cohort study in the United States reported no difference in retention between the two arms [23]. Studies from Haiti, South Africa and Lesotho were done in nonpregnant participants; however, none of the aforementioned studies stratified their participants by sexual behaviour or gender identity.

Given the paucity of data on same‐day ART in a non‐RCT setting, our study is among one of the first to illustrate its applicability and effectiveness when SDART is implemented in the real world. The promising findings obtained from this study suggest that immediate ART initiation, including SDART, can be done safely. Moreover, the simplified screening and laboratory algorithms can ensure that our model can be adaptable and generalizable to various places, such as those in resource‐constrained settings. Further, the results were stratified by gender identity and sexual behaviours. We showed that SDART initiation hub model did not negatively impact our study population, including those who were sexual minorities. Considering that MSM and TGW contributed to almost half of all new HIV infections in Thailand and are considered as key populations by UNAIDS [31], the SDART initiation hub model has the potential to help control the HIV epidemic. Nevertheless, more research must be done to examine the practicality of different SDART models that are context‐dependent and optimal for diverse populations. Also, longitudinal studies in various populations and settings are still needed to assess the influence of SDART on retention.

Several challenges and limitations should be acknowledged. One of the crucial limitations of a hub model is that data on long‐term outcomes, including times at which VL is tested, depend solely on the referred sites. This may skew the perceptions of the effectiveness of the model regarding VL suppression. According to the national guidelines, VL should be assessed every 6 months within the first year of ART initiation [25]. In 2018, 81.2% of the expected number of clients received a VL test, and 97.0% of these were virally suppressed [18]. Our cohort was reported to have 78.5% of the expected VL tested, and 94.6% of these were virally suppressed. To accurately evaluate the VL outcome of SDART, more efforts need to be made in increasing access to VL monitoring. The clients at TRC‐AC were also relatively healthy compared to other hospitals in Thailand, where clients presented with a median CD4 count of 125 cells/mm^3^ [32]. Thus, the lack of clinical practice guidelines in same‐day treatment may hamper the physician's willingness and confidence to start treatment upon the day of HIV diagnosis in a hospital setting due to concerns of potential illnesses associated with ART initiation. Nevertheless, our data were used to guide changes in Thailand's HIV Treatment and Prevention National Guidelines [33].

## 5 CONCLUSIONS

The SDART initiation hub model is acceptable and feasible, with the potential for scalability. The simplified and nonspecialized method of initiating treatment in the model also allows its applicability in broad settings.

## COMPETING INTERESTS

All authors declare no competing interests related to this work.

## AUTHORS’ CONTRIBUTIONS

PP, NP, PS, NT, TP, ST and RR designed the study. PS drafted the study protocol. PS, NP, RR and PP designed the analysis. PS, ST and PP secured ART used in the study. NP and PP secured funding for the study. NT, PS, TP, SA, SL, PJ, CP and PP conducted the study and collected the data. SA, SL, ON and RR monitored the accuracy of the data. PS coordinated the study, oversaw data management and wrote the first draft. PS, ON and RM analyzed the data with input from NP and PP. NP, RR and PP reviewed, revised and provided feedback on the draft. PS revised the final draft. The final draft was reviewed and approved by all authors.

## FUNDING

LINKAGES, a 5‐year cooperative agreement (AID‐OAA‐A‐14‐00045), is led by FHI 360 in partnership with IntraHealth International, Pact and the University of North Carolina at Chapel Hill.

## DISCLAIMER

The contents are the responsibility of the LINKAGES project and do not necessarily reflect the views of USAID, PEPFAR or the United States Government.

## APPENDIX

 

**Table A1:** Retention and viral suppression at 3, 6 and 12 months after ART initiation among all HIV‐positive clients

	Total	Heterosexual	MSM	TGW	*p*‐Value
**Clients on ART for 3 months**					
*n*	2427	679	1636	112	
Retained in care	1520 (62.6%)	361 (53.2%)	1085 (66.3%)	74 (66.1%)	0.785
Lost to follow‐up	311 (36.6%)	311 (45.8%)	541 (33.1%)	37 (33.0%)	<0.001
Discontinued ART	13 (0.5%)	5 (0.7%)	7 (0.4%)	1 (0.9%)	0.254
Death	5 (0.2%)	2 (0.3%)	3 (0.2%)	0(0.0%)	0.694
**Clients on ART for 6 months**					
*n*	2422	677	1633	112	
Retained in care	1428 (59.0%)	336 (49.6%)	1021 (62.5%)	71 (63.4%)	0.792
Lost to follow‐up	979 (40.4%)	335 (49.5%)	603 (36.9%)	41 (36.6%)	<0.001
Discontinued ART	14 (0.6%)	6 (0.9%)	8 (0.5%)	0 (0.0%)	0.252
Death	1 (0.0%)	0 (0.0%)	1 (0.1%)	0 (0.0%)	>0.99
**Clients on ART for 12 months**					
*n*	2421	677	1632	112	
Retained in care	1429 (59.0%)	330 (48.7%)	1028 (63.0%)	71 (63.4%)	0.329
Loss to follow‐up	983 (40.6%)	343 (50.7%)	599 (36.7%)	41 (36.6%)	<0.001
Discontinued ART	8 (0.3%)	4 (0.6%)	4 (0.2%)	0 (0.0%)	0.238
Death	1 (0.0%)	0 (0.0%)	1 (0.1%)	0 (0.0%)	>0.99
**Viral suppression**					
*n*	2427	679	1636	112	
Overall viral suppressed	1214 (50.0%)	292 (43.0%)	863 (52.8%)	59 (52.7%)	0.69
Numbers of VL testing (within the first year)	1297 (53.4%)	309 (45.5%)	923 (56.4%)	65 (58.0%)	
Viral suppressed (within the first year)	1197 (49.3%)	288 (42.4%)	850 (52.0%)	59 (52.7%)	>0.99
Viral suppressed (within the first year and among VL‐tested clients	1197/1297 (92.3%)	288/309 (93.2%)	850/923 (92.1%)	59/65 (90.8%)	
**Duration from ART initiation to viral suppression (days)**					
*n*	1258	302	895	61	
Median (IQR)	192 (157–252)	192.5 (153–252)	190 (157–249)	221 (160–282)	

Abbreviations: ART, antiretroviral therapy; IQR, interquartile range; MSM, men who have sex with men; TGW, transgender women; VL, viral load.

## Data Availability

The authors confirm that the data supporting the findings of this study are available within the article and its supplementary material.
